# Quantitative real-time imaging of intracellular FRET biosensor dynamics using rapid multi-beam confocal FLIM

**DOI:** 10.1038/s41598-020-61478-1

**Published:** 2020-03-20

**Authors:** James A. Levitt, Simon P. Poland, Nikola Krstajic, Karin Pfisterer, Ahmet Erdogan, Paul R. Barber, Maddy Parsons, Robert K. Henderson, Simon M. Ameer-Beg

**Affiliations:** 1 Microscopy Innovation Centre, Guy’s Campus, Kings College, London, SE1 1UL UK; 2 Richard Dimbleby Laboratories, School of Cancer and Pharmaceutical Sciences, Guy’s Campus, Kings College London, London, SE1 1UL UK; 3 Institute for Microelectronics and Nanosystems, School of Engineering, College of Science and Engineering, University of Edinburgh, Edinburgh, EH9 3FB UK; 4 Randall Centre for Cell and Molecular Biophysics, Guy’s Campus, Kings College, London, SE1 1UL UK; 5 UCL Cancer Institute, Paul O’Gorman Building, University College London, London, WC1E 6DD UK

**Keywords:** Biological fluorescence, Imaging and sensing

## Abstract

Fluorescence lifetime imaging (FLIM) is a quantitative, intensity-independent microscopical method for measurement of diverse biochemical and physical properties in cell biology. It is a highly effective method for measurements of Förster resonance energy transfer (FRET), and for quantification of protein-protein interactions in cells. Time-domain FLIM-FRET measurements of these dynamic interactions are particularly challenging, since the technique requires excellent photon statistics to derive experimental parameters from the complex decay kinetics often observed from fluorophores in living cells. Here we present a new time-domain multi-confocal FLIM instrument with an array of 64 visible beamlets to achieve parallelised excitation and detection with average excitation powers of ~ 1–2 μW per beamlet. We exemplify this instrument with up to 0.5 frames per second time-lapse FLIM measurements of cAMP levels using an Epac-based fluorescent biosensor in live HeLa cells with nanometer spatial and picosecond temporal resolution. We demonstrate the use of time-dependent phasor plots to determine parameterisation for multi-exponential decay fitting to monitor the fractional contribution of the activated conformation of the biosensor. Our parallelised confocal approach avoids having to compromise on speed, noise, accuracy in lifetime measurements and provides powerful means to quantify biochemical dynamics in living cells.

## Introduction

Quantitative imaging of the spatiotemporal dynamics of intracellular protein-protein interactions is challenging but essential for understanding the signalling processes that underpin cell behaviour. Developments in fluorescence microscopy and biosensors incorporating fluorescent proteins are making it possible to follow and quantify intracellular signalling events with high spatiotemporal accuracy in living specimens. Fluorescence lifetime imaging (FLIM) is a well-established, robust technique for quantitative intracellular measurements of protein-protein interactions by Förster resonance energy transfer (FRET)^1–4^, and highly photon efficient FRET measurements are achieved using FLIM with time-correlated single photon counting (TCSPC)^5^. In order to follow intracellular interactions using FLIM of biosensors based on FRET, measurements must often be made on the timescale of seconds, thus it is highly desirable to increase FLIM frame rates^6^ whilst preserving the spatial and temporal accuracy and retaining a modest light dose at the sample. Time-domain widefield FLIM based on gated cameras^7^, optical intensifiers^8–10^, and more recently TCSPC detection using crossed delay line anode detection^11^ and single photon avalanche diode (SPAD) arrays^12^, have been demonstrated with high frame rates. Commercial systems offering single beam scanning methods using fast digitization or TCSPC detection with short dead times allowing for higher photon counting rates^13,14^ are now also available. Multifocal excitation with widefield detection^15^, and TCSPC detection using a multi-anode PMT detector^16,17^, techniques that offer optical sectioning capabilities, have also been presented. Our lab has previously shown the use of 64-beam parallelised multi-focal multiphoton FLIM (MM-FLIM) with a SPAD array detector to measure intracellular FRET^18–20^, and also for simultaneous multi-plane volumetric imaging^21^.

Despite fast acquisition rates the number of photons that can be detected is also limited practically by the photophysics of the fluorophores used for labelling and the labelling density. High frame rate scanning FLIM can be combined with methods for rapid fluorescence lifetime estimation^20,22–24^, along with non-fitting methods for quantifying FRET^10,25,26^. The low number of photons available in a short acquisition means that resolving multi-exponential decays is often impractical, and this reduces the ability to separate and measure fractional contributions of donor fluorophores with differing levels of FRET interaction in a local environment, for example. For high frame-rate imaging which retains the benefits of beam scanning time-domain TCSPC FLIM: high signal to noise; excellent photon efficiency; high temporal and spatial accuracy; and optical sectioning capabilities, parallelisation of the excitation and detection is extremely beneficial. Parallelisation of the excitation in particular, allows for longer total pixel dwell times for a given frame rate compared to single beam methods, with the potential for lower peak laser powers per beam at the sample to yield a comparable number of photons.

Fluorescent biosensors with an optical readout based on FRET have been used to detect the second messenger cyclic adenosine monophosphate (cAMP)^27–31^, which plays a role in many cellular processes including adhesion. These biosensors, based on exchange protein activated by cAMP (Epac), an effector that mediates cAMP signalling, have undergone significant optimisation in recent years^29,30^. A palette of fluorescent biosensors based on Epac1 for detection of intracellular cAMP was recently presented^28^, including several constructs with a large dynamic range for FRET specifically optimised for FLIM, with the optimised cyan fluorescent protein, mTurquoise2 (mTurq2)^32–34^ as a donor and tandem dark Venus (td^d^Venus) acceptors which minimise the possibility of bleed-through of the acceptor emission into the donor detection channel.

In this work we present time-lapse TCSPC-FLIM imaging up to 0.5 frames per second (fps) using a multibeam parallelised array scanning TCSPC FLIM system with one-photon visible excitation, a single x-y tip/tilt scanning mirror and confocal detection. We quantified the evolution of the activated population of an Epac-based FRET biosensor for cAMP^28^ in living HeLa cells stimulated by forskolin and 3-isobutyl-l-methyl-xanthine (IBMX) with high spatiotemporal accuracy. Scanned arrays of excitation beamlets generated fluorescence beamlets from labelled living cells, which were imaged onto a single photon avalanche diode (SPAD) array with on-board time-to-digital converters (TDC) for each SPAD giving picosecond time resolution^35^. The microscope offers 300 nm lateral and 600 nm axial resolution and each SPAD also acts as a confocal pinhole to reject out of focus light. Importantly, the parallelised excitation permitted low average powers of ~ 1 μW per beamlet, reducing the probability of photodamage to the sample during imaging whilst retaining the spatiotemporal resolution necessary to capture the sample dynamics.

We used time-dependent phasor plots^36^ in a feed-forward manner to determine parameters for three-exponential global fits to the data from which we generated movies of the spatial variation of activated biosensor molecules following stimulation. The measured dynamic changes in E_FRET_ following stimulation occurred on a timescale of a few seconds. Although it is below the maximum achievable frame rate of our system, we chose an acquisition rate of 0.5 fps for time-lapse measurements, as it is commensurate with the timescale of the process under study. The measured fluorescence decays from the biosensor were multi-exponential and the FRET readout shows that local cAMP levels typically increase to a maximum within ~ 40 s before decreasing towards pre-stimulated values. Therefore, our fast confocal FLIM system, which benefits from both parallelised excitation and detection offers an effective way to measure the spatiotemporal variation in intracellular FRET efficiency, whilst retaining a low light dose at the sample.

## Results

### Time-lapse FLIM of live HeLa cells expressing empty vector mTurq2

To demonstrate the applicability of multi-confocal FLIM in cell biological imaging we first measured live HeLa cells expressing empty vector mTurq2 as a control. We observed homogeneous labelling of the cells, and data clusters in phasor plots **(**Supplementary movies 1 & 2**)** invariant in time and lying on the universal circle, indicative of a monoexponential decay, both prior to and after stimulation. We obtained fluorescence lifetimes, *τ*_control_ = 3.92 ± 0.08 ns (n = 16) from monoexponential fits to the data (5 × 5 pixel binning), in excellent agreement with the literature value^33^. This value was used to calculate E_FRET_ for subsequent biosensor measurements. The imaging parameters were 232 × 232 pixels with 40 µm × 40 µm field of view.

### Time-lapse FLIM of live HeLa cells expressing mTurq2-Epac1-td^d^Venus biosensor

**Figure 1:**
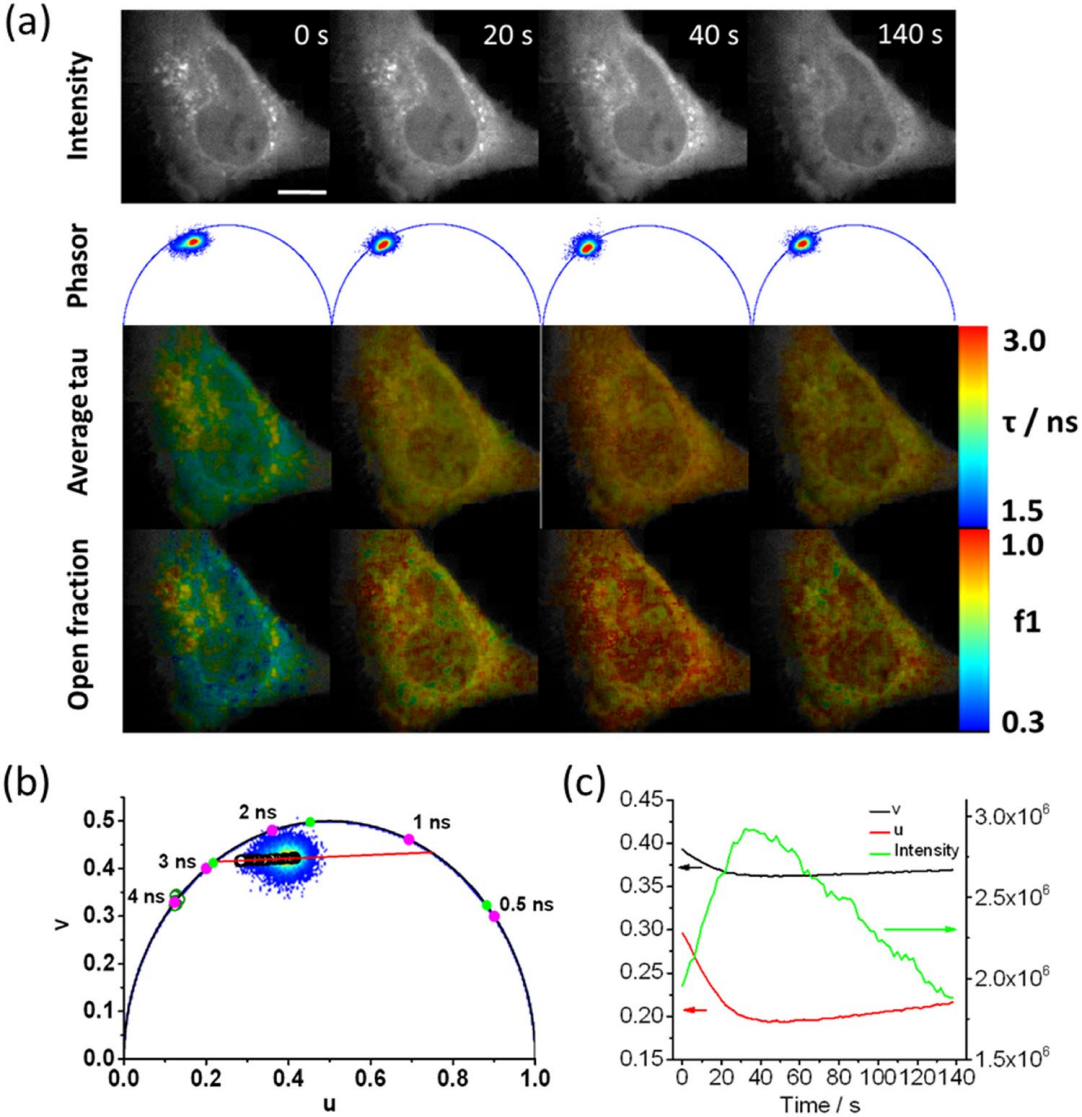
Fluorescence lifetime imaging of HeLa cells expressing an mTurq2-Epac1-td^d^Venus biosensor. (**a**) Representative fluorescence intensity, corresponding phasor plots, average fluorescence lifetime FLIM images, and images of the fractional contribution of the open “activated” biosensor of a HeLa cell expressing mTurq2-Epac1-td^d^Venus biosensor at 4 time points following stimulation with forskolin (25 µM) and IBMX (100 µM). Images are 232 × 232 pixels with a field of view of 40 µm × 40 µm, an acquisition time of 2 s per frame and pixel binning of 7 × 7 pixels. (**b**) A representative phasor plot showing the cluster of data points prior to stimulation and the time-dependent position of the data cluster after stimulation (open black circles). The trajectory of the phasor as a function of time after stimulation can be well fitted to a straight line (solid red line). The phasor positions from control measurements from HeLa cells expressing empty vector mTurq2 (open green circles) lie on or very close to the universal circle. Three fluorescence lifetime contributions calculated from multi-image global fits to time-lapse imaging data (solid green circles) are shown along with reference calculated positions for several monoexponential fluorescence lifetimes (filled magenta circles). (**c**) Plots of u, v from the phasor plots, and total intensity values from the time-lapse measurements.

**Figure 2:**
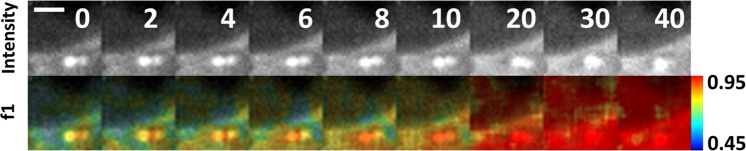
Single SPAD images of HeLa cells expressing an mTurq2-Epac1-td^d^Venus biosensor following stimulation. Images of fluorescence intensity (upper panel) and fractional contribution of the open conformation, f1, of the biosensor (lower panel) for 9 time points following stimulation. Images are from a single SPAD detector and show an example of spatiotemporal dynamics in perinuclear bright punctate labelling. The field of view for each image (30 × 30 pixels) is 5 µm × 5 µm, The FLIM acquisition was a total of 6 s per image, with time intervals of 2 s. Scale bar 2 µm.

We imaged HeLa cells transfected with a mTurq2-Epac1-td^d^Venus biosensor (232 × 232 pixels with 40 µm × 40 µm field of view) in time-lapse FLIM acquisitions prior to and following stimulation with forskolin (25 µM) and IBMX (100 µM)^28^ (Fig. 1a). The biosensor was present throughout the cell with punctate labelling primarily in the perinuclear region. A small increase in fluorescence intensity was observed following stimulation at t = 0 s, before a decrease to pre-stimulation values for the total fluorescence intensity at longer time points, when the contrast in intensity between the punctate regions and the rest of the cell was greatly reduced. The corresponding phasor plots (Fig. 1a) demonstrated that prior to stimulation, the cluster of data points was located inside the universal circle. The position of the data cluster for the cells expressing mTurq2-Epac1-td^d^Venus was invariant during pre-stimulation time-lapse FLIM measurements recorded with an acquisition time of 2 s per frame **(**Supplementary movie 3**)**, but moved towards, and in some cases reached, the edge of the universal circle during the post-stimulation time-lapse measurements **(**Supplementary movie 4**)**. This is indicative of emission from multiple fluorescent species/states prior to stimulation moving towards emission from a single fluorescent species (monoexponential decay with the data cluster lying on the universal circle) following stimulation. We assert that this is indicative of a distribution of activated “open” and non-activated “closed” conformations of biosensor molecules within each pixel of the image. The conformational change of the mTurq2-Epac1-td^d^Venus biosensor from closed to open upon stimulation increases the distance between the mTurq2 donor and the dark Venus acceptors, reducing the FRET efficiency and increasing the donor fluorescence lifetime^28^. This occurs in a binary manner, i.e. the biosensor adopts only open and closed conformations rather than a continuum of conformations between open and closed. In all cases the trajectory of the data clusters across the universal circle could be fitted to a straight line **(**Fig. 1b**)**. This is typically indicative of two distinct emitting species with fixed FRET efficiencies, with the position of the points representing the weighting of each of the decay components^36^. We note that the cluster of data points typically becomes more circular as the time-lapse measurements progress **(**Supplementary movie 4**)** indicating a greater degree of homogeneity between pixels in the amount of open and closed biosensors in response to stimulation, which propagated rapidly throughout the cell. The intersection of the extrapolated straight line fit to the biosensor data with the left hand side of the universal circle provided the fluorescence lifetime of the open conformation, *τ*_open_ = 2.93 ± 0.11 ns (n = 15), which was shorter than the control mTurq2 values (Fig. 1b, open green circles), corresponding to a FRET efficiency for the open conformation, E_FRET,open_ = 25.3% ± 3.2%, in reasonable agreement with the published FRET efficiency of the stimulated open conformation (19.4%)^28^. However, from the intersection of the extrapolated linear fit with the right hand side of the universal circle the fluorescence lifetime of the closed conformation, *τ*_closed_, were as short as 0.88 ns, corresponding to E_FRET,closed_ = 77.6% which even considering anomalously high FRET efficiencies from multiple acceptors^37^, is deemed too high to be physically realistic and does not correspond to the reported basal unstimulated E_FRET_ = 55.3%^28^. We therefore propose that there is an additional short lifetime component in addition to the two longer lifetime components corresponding to the open and closed conformations of the biosensor. Possible sources of the short lifetime component are emission from the tandem dark Venus acceptors, given that it has previously been shown that REACh acceptors fluoresce with short (<500 ps) lifetimes^38^, or contributions from non-isotropic orientations of the large fluorescent protein labels on the biosensor on the timescale of the fluorescence^39^. Assuming well-defined contributions from the open and closed forms of the biosensor with an additional short lifetime component, a three-exponential model is necessary to fit the data. We therefore performed three-exponential multi-image global fits to the data (7 × 7 pixel binning) (see Methods) with the longest fluorescence lifetime, corresponding to *τ*_open_, fixed at 2.93 ns. These fits were used to quantify the spatiotemporal variation in the population of the open conformation of the biosensor as a function of time during the experiment (Fig. 1a). The total fluorescence intensity from the cell peaks after ~40 s, before decreasing to approximately the initial value after 140 s. Average lifetime FLIM images show an overall reduction in fluorescence lifetime at images between 40 s and 140 s time points (Fig. 1a), as expected. However, images of the contribution of the open configuration from the tri-exponential fits to the data give a measure of the spatiotemporal distribution of activated, open form of the biosensor. We observed data clusters that moved back towards the initial, pre-stimulation position although did not fully return to this baseline position during the course of the measurement despite the reduction in fluorescence intensity after the initial rise **(**Fig. 1c**)**. This is indicative of photobleaching rather than just an increased FRET efficiency over the course of the measurements. Cells expressing the mTurq2-Epac1-td^d^Venus biosensor typically exhibited a punctate distribution of the fluorescence with translational motion of the puncta observed on the timescale of the experiment **(**Supplementary movies 3 & 4**)**. We measured the fractional contribution of the open conformation of the biosensor in these puncta (~1 μm diameter) in our time-lapse images (Fig. 2**)**. In these images the two dynamic puncta were initially separated by ~ 1 μm, but moved to overlap over a period of 40 s. Our FLIM images show clear differences in the fractions of activated biosensor present in each of these puncta at several time points during the acquisition.

## Discussion

By imaging using a multibeam confocal TCSPC FLIM microscope with visible excitation we were able to quantify the fraction of activated cAMP biosensor molecules in live HeLa cells as a function of time by measuring intracellular changes in FRET following stimulation using forskolin and IBMX. The rapid and spatially variant response of the FRET biosensors to stimulation in single cells was particularly challenging to quantify, due to the presence of multiexponential fluorescence decays. In most cases the population of activated biosensor molecules (open conformation) reached a maximum value within ~ 60 s.

Phasor plots provide an excellent means to inspect the FLIM data to ensure that any model used to fit the fluorescence decays is valid. We used the phasor plots to determine that at least three exponentials were needed to accurately describe the live cell data. Additionally, the phasor data show that the decrease in intensity at long times following stimulation is due in part to an increase in FRET and may also be attributed to photobleaching due to prolonged exposure of the cell to the excitation laser. Feeding forward from the phasor plots, quantification of the spatially heterogeneous and time-varying distributions of active and inactive biosensor molecules was afforded by the calculation of fractional contributions of each fluorescence lifetime component as determined by pixel-wise fitting of a three-exponential model to the data with each of the three fluorescence lifetimes determined by a multi-image global fit to the data.

We have shown that one-photon parallelised excitation and detection using visible light and confocal detection using the active area of individual SPADs as pinholes is a viable and elegant solution to the problem of long acquisition times in traditional TCSPC FLIM. In our arrangement the power per beamlet is comparable to standard single beam scanning systems which minimises the potential for photobleaching and generation of phototoxic species. Analysis of the time-resolved TCSPC data using a combination of phasor plots and multi-exponential decay fits has enabled us to confirm that the time-evolving fluorescence from the biosensor following activation can be described by varying fractional contributions of open and closed conformations. By comparison, such measurements using frequency domain FLIM would typically require multiple sequential measurements with varying modulation frequencies, and conventional single beam TCSPC measurements of these dynamics would require higher excitation powers to reach a comparable frame rate and spatiotemporal resolution. Our acquisition rates for FLIM were not the maximum achievable using this system but were chosen as an excellent compromise between the time necessary to capture the biological dynamics and ensure a sufficient number of photons were collected for analysis without the need to increase the excitation intensity, therefore minimising photobleaching during the acquisition period. It is the combination of this relatively high frame rate TCSPC detection with Nyquist spatial sampling that permits us to follow such dynamic intracellular processes in real time.

## Methods

### Multibeam scanning confocal FLIM microscopy

**Figure 3:**
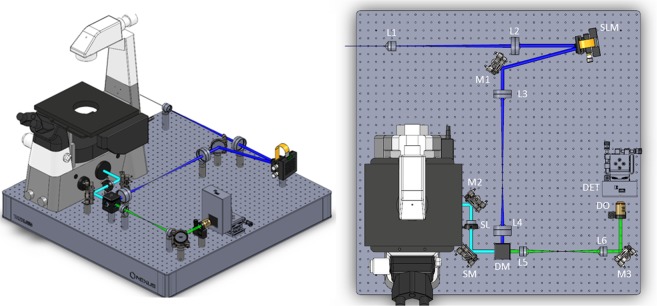
Schematic optical arrangement for confocal multibeam FLIM microscopy. M1-M3 – Protected silver coated steering mirrors; L1 – f = 40 mm achromatic doublet lens; PH – 25 μm diameter pinhole; L2 – f = 250 mm achromatic doublet lens; SLM – LCOS spatial light modulator; L3 - f = 300 mm achromatic doublet lens; L4 - f = 150 mm achromatic doublet lens; DM – dichromatic mirror; SM – tip/tilt scan mirror; SL – f = 100 mm achromatic doublet scan lens; L5 - f = 100 mm achromatic doublet lens; L6 - f = 200 mm achromatic doublet lens; M3 – piezo fine alignment steering mirror; DO–4x NA0.13 objective lens; DET – SPAD array detector.

The schematic optical arrangement of the multibeam scanning confocal microscope is shown in Fig. 3. For excitation of mTurq2, a 435 nm beam was generated by frequency-doubling a femtosecond mode-locked Ti:Sapphire laser (Mai Tai, Newport Spectra Physics, USA) in a custom motorised SHG system (UHG-Series, GWU, GmbH) and passed through a 25 mm path length SF57 glass rod (Schott Glass, GmbH), cut at Brewster’s angle for 488 nm and polished at each end (IC Optical Systems Ltd, UK), to temporally broaden the pulse to avoid unnecessarily high laser intensities at the sample which may cause non-linear effects. A spatial filter and beam expander consisting of an achromatic doublet lens (f = 40 mm, AC254-040, Thorlabs Ltd, UK), a 25 μm diameter pinhole (P25C, Thorlabs Ltd, UK) and an achromatic doublet lens (f = 250 mm, AC254-250, Thorlabs Ltd, UK) was used to improve the beam quality after second harmonic generation before generation of an array of beamlets using a liquid crystal on silicon spatial light modulator (LCOS-SLM, P512, Meadowlark, USA). The active area of the SLM (7.68 mm × 07.68 mm) was illuminated by a circular beam expanded to a diameter ~ 6 mm (1/e^2^). Using a doubly weighted Gerchberg-Saxton (DWGS) iterative phase retrieval algorithm^40^, the appropriate holographic phase pattern was calculated and projected onto the SLM, which was positioned in the Fourier plane, the required laser beamlets were generated experimentally at the focal plane of a lens. The beamlet array was reflected off a dichromatic reflector (Di03-R442-t3-25 × 36, Semrock, USA) and conjugated to a single tip/tilt x-y piezo scanning mirror (S334.2 SD, Physik Instrumente, Germany). The use of this tip/tilt mirror provided a compact and efficient optical arrangement by avoiding the necessity for either close coupled galvanometer mirrors or an afocal relay. The beamlets were scanned across the sample through a 40× NA1.3 microscope objective (Nikon Instruments (UK) Ltd) with an effective pixel size of 156 nm. The resultant fluorescence was descanned, separated from the excitation light at the dichromatic mirror and relayed to the pupil of an auxiliary objective (4× NA 0.13 microscope objective (Nikon Instruments (UK) Ltd)) to focus each beamlet onto a separate SPAD detector of a Megaframe 32 camera^35^. The measured uniformity in intensity across the 8 × 8 sub-array of the MF32 detector was typically >80%. For display purposes a flat field correction, derived from imaging a fluorescent slide (Chroma, USA) was applied to intensity images. The calculated fluorescence spot size was 10 µm (1/e^2^) such that each 6 µm diameter SPAD is effectively a confocal pinhole of ~0.6 Airy units. The beamlet array alignment, scanning, and data collection was controlled via custom LabVIEW Virtual Instruments. The instrument response function (IRF) for each SPAD was measured using a fluorescein in saturated NaI aqueous NaOH solution^41^, and the time-resolved data were iteratively re-convolved for the multi-exponential fitting. The photon arrival times from the TCSPC data were histogrammed to generate fluorescence decays for all pixels of the image, and data were corrected for differential and integral non-linearity^42^. It should be noted that as the system operates as an array of individual confocal FLIM microscopes, each scanning a small sub-region of the total field of view, the power per beamlet is comparable to that of the single beam in a standard confocal microscope and there is no associated penalty in terms of photobleaching due to increased power. The microscope was equipped with a live cell imaging chamber (Solent Scientific Ltd, UK) enabling imaging at 37 °C.

### Characterisation of spatial and temporal performance

We calibrated the performance of the system using 0.2 µm fluorescent microspheres (TetraSpeck^TM^, ThermoFisher Scientific, UK). The microspheres were excited by scanning an 8 × 8 beamlet array at 435 nm through a 100× NA1.45 objective (CFI Plan Apochromat Lambda 100× Oil, Nikon, UK) on an inverted microscope (Nikon Ti Eclipse, Nikon, UK) equipped with a perfect focus system. The effective pixel size was 62.5 nm in the x-y plane. The lateral and axial resolution was determined to be 302 ± 29 nm (s.d.) and 600 ± 96 nm (s.d.), respectively, from Gaussian fits to the data (Supplementary Fig. 1). Photon arrival times at the MF32 detector were calculated relative to a trigger (sync) signal from laser pulses incident on an optical constant fraction discriminator (OCF-401, Becker & Hickl, GmbH)) at the repetition rate of the laser, 80 MHz, which is sent to the MF32 detector. The temporal characterisation of the system was evaluated by adding a fixed delay of 5 ns to the photon arrival times by decreasing the sync cable (RG-174 type) length by 1 m. From measurements of the peak position and the decay curve from the sodium fluorescein solution we calculated the temporal bin size to be 57 ps. Representative intensity images of cells expressing empty vector mTurq2 and mTurq2-Epac1-td^d^Venus and fluorescence decays from 5 × 5 pixel regions of the images are shown in Supplementary Fig. 2.

### Cell culture and reagents

HeLa cells were cultured in Dulbecco’s Modified Eagles Medium supplemented with L-glutamine, fetal bovine serum (10%) and antibiotics (PenStrep) at 37 °C in a 5% CO_2_ atmosphere. Cells were transiently transfected with a high-affinity Epac biosensor construct with an mTurq2 donor and a tandem dark Venus acceptor (H189)^28^ 24–36 h prior to imaging using Effectene (Qiagen, Hilden, Germany). Cells were seeded in 8-well chamber slides (µ-Slide 8 Well Glass Bottom, Ibidi, GmbH) and the medium was supplemented with HEPES and imaged at 37 °C. As a control for the lifetime of the mTurq2 (donor) alone, cells expressing empty vector mTurq2 were imaged under the same conditions as the cells expressing the biosensor. Intracellular cAMP levels were increased by stimulation using forskolin (25 µM) and IBMX (100 µM).

### FLIM data analysis

All fitting of the FLIM data was performed in the TRI2 analysis enviroment^43^. Subsequent additional data analysis was performed using Origin 2015 (OriginLab). Phasor plots were generated by taking Fourier transforms of the data^36^. In the phasor representation of the data a fitting model is not required and each pixel element in the time-resolved images is represented by a single point on the phasor plot. The fluorescence lifetime measured from the cells transfected with empty vector mTurq2 was invariant in time both prior to and after stimulation and were fitted using a monoexponential decay model for each frame (232 × 232 pixels) with 5 × 5 pixel binning. Analysis of time-dependent trajectories of data clusters on the phasor plots from time-lapse FLIM experiments were used to define parameters for pixel-wise fitting to an exponential decay model using a Levenberg-Marquardt fitting procedure and a multi-image global analysis routine. Briefly, the time-resolved data from the time-lapse imaging were processed simultaneously using a multi-image three-exponential global fit with one fluorescence lifetime component, *τ*_1_ = 2.93 ns, fixed corresponding to the point at which a linear fit to the trajectory of data cluster in the phasor plots intercepts the universal circle. Other lifetime components were allowed to vary but were assumed to be constant across the whole dataset. Component amplitudes were allowed to vary at every image pixel independently. The intensity-weighted fractional contributions, *f*_i_ (where i = 1, 2, 3) with fluorescence decay amplitudes, *A*_*i*_, of each of the lifetime components, *τ*_1_ = 2.93 ns, *τ*_2_ = 1.65 ns, *τ*_3_ = 0.55 ns (Fig. 1b, closed green circles), were then calculated using Eq. S1. From these fits time-lapse maps of the spatiotemporal evolution of the fractional contribution of open conformation of the biosensor, *f*_1_, were generated by pixel-wise three-exponential fits to the data in each frame using the fixed *τ*_i_ values, and 7 × 7 pixel binning.

S1 $${f}_{i}=\frac{{A}_{i}{\tau }_{i}}{{\sum }_{j}{A}_{j}{\tau }_{j}}$$

### Empty vector mTurq2 control data

We first generated phasor plots of the data using TRI2. In phasor plots each pixel from the scanned image of the sample yields a single data point on the universal circle. The position of the data point (*u*, *v*) on the phasor plot is given by a Fourier transform of the time-resolved data (Eqs. S2, S3).

S2 $$u=\frac{{\int }_{0}^{\infty }\,I(t)\,\cos \,(\omega t)dt}{{\int }_{0}^{\infty }\,I(t)dt}$$

S3 $$v=\frac{{\int }_{0}^{\infty }I(t)\,\sin (\omega t)\,dt}{{\int }_{0}^{\infty }I(t)dt}$$

The fitted fluorescence lifetimes and the fractions of each components are related to u and v by Eqs. S4 and S5.

S4 $${u}_{ij}(\omega )=\sum _{k}\,\frac{{f}_{k}}{1+{(\omega {\tau }_{k})}^{2}}$$

S5 $${v}_{ij}(\omega )=\sum _{k}\,\frac{{f}_{k}\omega {\tau }_{k}}{1+{(\omega {\tau }_{k})}^{2}}$$

where *f*_*k*_ is the intensity weighted fractional contribution of species *k*, *τ*_*k*_ is the fluorescence lifetime of the species *k* and *ω* is the frequency of the measurement period.

In the case of the empty vector mTurq2 the resultant phasor plots showed a cluster of data points centred on the universal circle as expected for mTurq2 which has a reported monoexponential fluorescence decay of 4.0 ns in mammalian cells^33^. Pixel-wise Levenberg-Marquardt fitting of the data to a monoexponential model yielded good fits to the data. A series of time-lapse FLIM images was recorded with an acquisition time of 2 s per frame following the addition of forskolin (25 μM) and IBMX (100 μM). The position of the data cluster in the phasor plots remained invariant as a function of time both prior to and following stimulation **(**Supplementary movies 1 & 2**)**, although the fluorescence intensity decreased due to photobleaching.

### mTurq2-Epac1-td^d^Venus data - Calculating time-varying populations of open and closed form of biosensor

#### Phasor plots

We first generated phasor plots for the time-lapse image series recorded in with an acquisition time of 2 s for HeLa cells transfected with mTurq2-Epac1-td^d^Venus with and without stimulation by forskolin and IBMX. The cluster of data points on the phasor plot lies on the universal circle if the lifetime is monoexponential, i.e. only one fluorescent species being measured. In the case of no stimulation the position of the data cluster inside the universal circle in the phasor plot was invariant throughout the time-lapse experiment (70 frames acquisition). Following stimulation, the data cluster in the phasor plots traced out a trajectory from inside the universal circle towards the edge of the universal circle, which was fitted to a straight line that was extrapolated to intercept the universal circle at each end. In the simplest case the position of the data cluster along this chord gives the relative fractions of the two emitting species with the intercepts of the chord with the universal circle giving the fluorescence lifetimes of each of the emitting species (longer lifetime components on the left-hand side of the universal circle). We observed that the trajectory of the data clusters and the positions at which the chord met the surface of the universal circle did not correspond to published FRET efficiencies for the closed (high-FRET efficiency conformation) of the biosensor^28^.

#### Multi-exponential fits

Following the phasor analysis, we analysed the biosensor FLIM data using a three-exponential fitting model with multi-image global analysis in TRI2. In this approach global analysis is performed on all selected images in a data set, i.e. a time-lapse series, simultaneously. The fractional components of the open conformation were then calculated by a three-exponential fit to the data using the fixed fluorescence lifetimes obtained from the multi-image global analysis. Spatial binning of 7 × 7 pixels was used for the fitting. In the case of the images of small features (Fig. 2) recorded using a single SPAD (30 × 30 pixels) we used a rolling sum of three images from the time-lapse data (i.e. summing images 1 to 3, 2 to 4, 3 to 5….n-2 to n, with a total effective acquisition time of 6 s in 2 s intervals). This increased the quality of the fitting by increasing the number of photons per pixel without introducing significant motion blur. The fractional contributions of species, *f*_*i*_, (where *i* = 1, 2, 3) were then calculated and images of the fractional contribution of the open conformation of the biosensor were generated.

## Supplementary information

Supplementary Figures.

Supplementary movie 1.

Supplementary movie 2.

Supplementary movie 3.

Supplementary movie 4.
