# Supplementary material for: Quantitative real-time imaging of intracellular FRET biosensor dynamics using rapid multi-beam confocal FLIM
Source: Sci Rep. 2020 Mar 20;10:5146. doi: 10.1038/s41598-020-61478-1 (PMC7083966; doi:10.1038/s41598-020-61478-1)
Supplement: Supplementary file 1 — Supplementary Figures. [file 41598_2020_61478_MOESM1_ESM.pdf]

Quantitative real-time imaging of intracellular FRET biosensor dynamics using rapid multi-beam confocal FLIM

James A. Levitt[1,2], Simon P. Poland[2], Nikola Krstajic[3], Karin Pfisterer[4], Ahmet Erdogan[5], Paul R. Barber[6], Maddy Parsons[4], Robert K. Henderson[5] and Simon M. Ameer-Beg[2*]

[1]Microscopy Innovation Centre, Guy's Campus, Kings College, London, SE1 1UL, UK.

[2]Richard Dimbleby Laboratories, School of Cancer and Pharmaceutical Sciences, Guy's Campus, Kings College London, London, SE1 1UL, UK.

[3] School of Science and Engineering, Fulton Building, University of Dundee, DD1 4HN, UK.

[4]Randall Centre for Cell and Molecular Biophysics, Guy's Campus, Kings College, London, SE1 1UL, UK.

[5]Institute for Microelectronics and Nanosystems, School of Engineering, College of Science and Engineering, University of Edinburgh, EH9 3FF, UK.

[6]UCL Cancer Institute, Paul O'Gorman Building, University College London, London, WC1E 6DD, UK.

*Corresponding author. Email: simon.ameer-beg@kcl.ac.uk

Supplementary Information

Supplementary figures

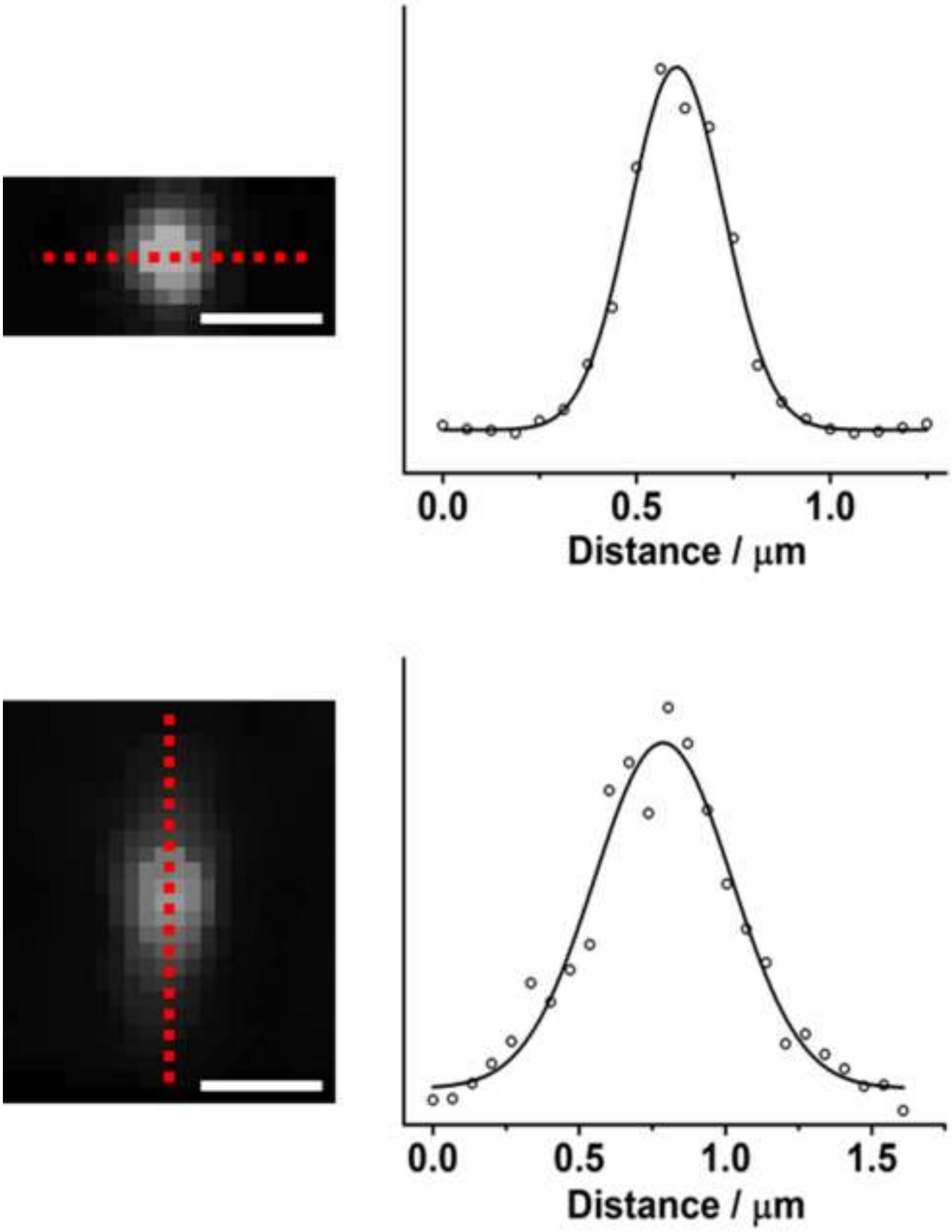

Supplementary figure 1: (a) Representative lateral (upper panel) and axial (lower panel) confocal measurements of 0.2 µm microspheres with excitation at 435 nm

and emission at 485 nm with Gaussian fits (black solid lines) to data points (open circles) from line profiles of the images. Scale bar 0.5 µm.

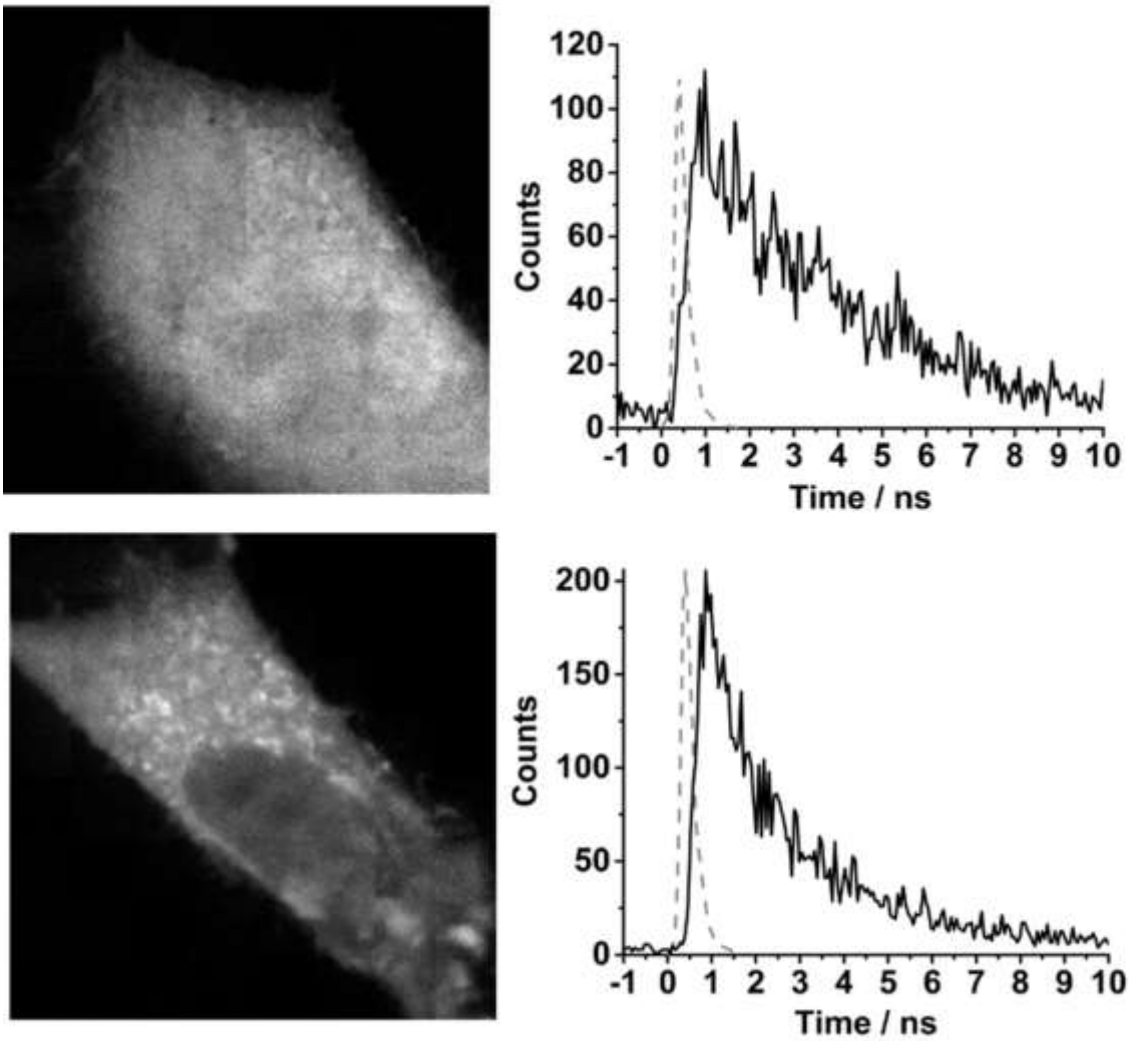

Supplementary figure 2

Fluorescence intensity and representative fluorescence decays (5x5 pixels) from HeLa cells expressing empty vector mTurq2 (Upper Panel) and mTurq2-Epac1-tdDVenus (lower Panel). Scale bar 5 µm.

Supplementary Movie Captions

Supplementary movie 1: FLIM images from a monoexponential fit (5x5 pixel binning) and phasor plots for a Hela cell expressing empty vector mTurq2 measured for 10 frames (acquisition time 2 s/frame) prior to addition of forskolin (25 µM) and IBMX (100 µM). Scale bar 5 µm.

Supplementary movie 2: FLIM images from a monoexponential fit (5x5 pixel binning) and phasor plots for a Hela cell expressing empty vector mTurq2 measured for 45 frames (acquisition time 2 s/frame) following the addition of forskolin (25 µM) and IBMX (100 µM). Scale bar 5 µm.

Supplementary movie 3: Intensity, phasor plots and images of the fractional contribution, f_1, of the open “activated” biosensor from tri-exponential fits to the FLIM data (7x7 pixel binning) for a Hela cell expressing mTurq2-Epac1-tdDVenus measured for 20 frames (acquisition time 2 s/frame) prior to addition of forskolin (25 µM) and IBMX (100 µM). Scale bar 5 µm.

Supplementary movie 4: Intensity, phasor plots and Images of the fractional contribution, f_1, of the open “activated” biosensor from tri-exponential fits to the FLIM data (7x7 pixel binning) for a Hela cell expressing mTurq2-Epac1-tdDVenus measured for 70 frames (acquisition time 2 s/frame) following the addition of forskolin (25 µM) and IBMX (100 µM). Scale bar 5 µm.
